# Supplementary material for: Towards a unified molecular mechanism for ligand-dependent activation of NR4A-RXR heterodimers
Source: eLife. 2026 Apr 30;14:RP106861. doi: 10.7554/eLife.106861 (PMC13132549; doi:10.7554/eLife.106861)
Supplement: Supplementary file 1. [file elife-106861-supp1.docx]

**gBlocks:**

**dNTD-RXRg (139-459):**

CTTAAGCTTGGTACCGAGCTCGATGTGTGCTATCTGTGGAGACAGATCCTCAGGAAAGCACTACGGGGTATACAGTTGTGAAGGCTGCAAAGGGTTCTTCAAGAGGACGATAAGGAAGGACCTCATCTACACGTGTCGGGATAATAAAGACTGCCTCATTGACAAGCGTCAGCGCAACCGCTGCCAGTACTGTCGCTATCAGAAGTGCCTTGTCATGGGCATGAAGAGGGAAGCTGTGCAAGAAGAAAGACAGAGGAGCCGAGAGCGAGCTGAGAGTGAGGCAGAATGTGCTACCAGTGGTCATGAAGACATGCCTGTGGAGAGGATTCTAGAAGCTGAACTTGCTGTTGAACCAAAGACAGAATCCTATGGTGACATGAATATGGAGAACTCGACAAATGACCCTGTTACCAACATATGTCATGCTGCTGACAAGCAGCTTTTCACCCTCGTTGAATGGGCCAAGCGTATTCCCCACTTCTCTGACCTCACCTTGGAGGACCAGGTCATTTTGCTTCGGGCAGGGTGGAATGAATTGCTGATTGCCTCTTTCTCCCACCGCTCAGTTTCCGTGCAGGATGGCATCCTTCTGGCCACGGGTTTACATGTCCACCGGAGCAGTGCCCACAGTGCTGGGGTCGGCTCCATCTTTGACAGAGTCCTAACTGAGCTGGTTTCCAAAATGAAAGACATGCAGATGGACAAGTCGGAACTGGGATGCCTGCGAGCCATTGTACTCTTTAACCCAGATGCCAAGGGCCTGTCCAACCCCTCTGAGGTGGAGACTCTGCGAGAGAAGGTTTATGCCACCCTTGAGGCCTACACCAAGCAGAAGTATCCGGAACAGCCAGGCAGGTTTGCCAAGCTGCTGCTGCGCCTCCCAGCTCTGCGTTCCATTGGCTTGAAATGCCTGGAGCACCTCTTCTTCTTCAAGCTCATCGGGGACACCCCCATTGACACCTTCCTCATGGAGATGTTGGAGACCCCGCTGCAGATCACCTGAGATCCACTAGTCCAGTGTGG

**RXRg-hinge-LBD (205-459):**

CTTAAGCTTGGTACCGAGCTCGATGAAGAGGGAAGCTGTGCAAGAAGAAAGACAGAGGAGCCGAGAGCGAGCTGAGAGTGAGGCAGAATGTGCTACCAGTGGTCATGAAGACATGCCTGTGGAGAGGATTCTAGAAGCTGAACTTGCTGTTGAACCAAAGACAGAATCCTATGGTGACATGAATATGGAGAACTCGACAAATGACCCTGTTACCAACATATGTCATGCTGCTGACAAGCAGCTTTTCACCCTCGTTGAATGGGCCAAGCGTATTCCCCACTTCTCTGACCTCACCTTGGAGGACCAGGTCATTTTGCTTCGGGCAGGGTGGAATGAATTGCTGATTGCCTCTTTCTCCCACCGCTCAGTTTCCGTGCAGGATGGCATCCTTCTGGCCACGGGTTTACATGTCCACCGGAGCAGTGCCCACAGTGCTGGGGTCGGCTCCATCTTTGACAGAGTCCTAACTGAGCTGGTTTCCAAAATGAAAGACATGCAGATGGACAAGTCGGAACTGGGATGCCTGCGAGCCATTGTACTCTTTAACCCAGATGCCAAGGGCCTGTCCAACCCCTCTGAGGTGGAGACTCTGCGAGAGAAGGTTTATGCCACCCTTGAGGCCTACACCAAGCAGAAGTATCCGGAACAGCCAGGCAGGTTTGCCAAGCTGCTGCTGCGCCTCCCAGCTCTGCGTTCCATTGGCTTGAAATGCCTGGAGCACCTCTTCTTCTTCAAGCTCATCGGGGACACCCCCATTGACACCTTCCTCATGGAGATGTTGGAGACCCCGCTGCAGATCACCTGAGATCCACTAGTCCAGTGTGG
